# Prognostic Outcomes in Acute Myocardial Infarction Patients Without Standard Modifiable Risk Factors: A Multiethnic Study of 8,680 Asian Patients

**DOI:** 10.3389/fcvm.2022.869168

**Published:** 2022-03-29

**Authors:** Gwyneth Kong, Nicholas W. S. Chew, Cheng Han Ng, Yip Han Chin, Oliver Z. H. Lim, Anand Ambhore, Gavin Ng, William Kong, Kian-Keong Poh, Roger Foo, James Yip, Tiong-Cheng Yeo, Adrian Fatt-Hoe Low, Chi-Hang Lee, Mark Yan-Yee Chan, Huay-Cheem Tan, Poay-Huan Loh

**Affiliations:** ^1^Yong Loo Lin School of Medicine, National University of Singapore, Singapore, Singapore; ^2^Department of Cardiology, National University Heart Centre, National University Health System, Singapore, Singapore

**Keywords:** hypertension, hyperlipidemia, diabetes, standard modifiable cardiovascular risk factors, prognostic outcomes, smoking, acute myocardial infarction

## Abstract

**Background:**

An increasing proportion of patients with acute myocardial infarction (AMI) are presenting without standard modifiable risk factors (SMuRFs) of hypertension, hypercholesterolemia, diabetes, and smoking, but with an unexpectedly increased mortality. This study examined the SMuRF-less patients presenting with AMI in a multiethnic Asian population.

**Methods:**

We recruited patients presenting with AMI from 2011 to 2021 and compared the prevalence, clinical characteristics, and outcomes of SMuRF-less and SMuRF patients. Multivariable analysis was used to compare the outcomes of 30-day cardiovascular mortality, all-cause mortality, readmission, cardiogenic shock, stroke, and heart failure. Kaplan–Meier curves were constructed for 30-day cardiovascular mortality, with stratification by ethnicity, gender and AMI type, and 10-year all-cause mortality.

**Results:**

Standard modifiable risk factor-less patients, who made up 8.6% of 8,680 patients, were significantly younger with fewer comorbidities that include stroke and chronic kidney disease, but higher rates of ventricular arrhythmias and inotropic or invasive ventilation requirement. Multivariable analysis showed higher rates of cardiovascular mortality (HR 1.48, 95% CI: 1.09–1.86, *p* = 0.048), cardiogenic shock (RR: 1.31, 95% CI: 1.09–1.52, *p* = 0.015), and stroke (RR: 2.51, 95% CI: 1.67–3.34, *p* = 0.030) among SMuRF-less patients. A 30-day cardiovascular mortality was raised in the SMuRF-less group, with similar trends in men, patients with ST-segment elevation myocardial infarction (STEMI), and the three Asian ethnicities. All-cause mortality remains increased in the SMuRF-less group for up to 5 years.

**Conclusion:**

There is a significant proportion of patients with AMI without standard risk factors in Asia, who have worse short-term mortality. This calls for greater focus on the management of this unexpectedly high-risk subgroup of patients.

## Introduction

The control of cardiovascular risk factors is paramount in the prevention of adverse cardiovascular outcomes including stroke and acute myocardial infarction (AMI) (1). Hence, early identification and intervention of standard modifiable risk factors (SMuRFs), such as hypercholesterolemia (2), hypertension (3), smoking (4), and diabetes mellitus (5), are essential in reducing the atherosclerotic cardiovascular disease risk of all individuals (6–9) and the prevention of cardiovascular disease (10). The recent studies have shown a growing proportion of patients without SMuRF [termed SMuRF-less (11)] who were previously asymptomatic, presenting with ST-segment elevation myocardial infarction (STEMI) (12). Their prevalence among patients presenting with STEMI has increased over the past decade from 13% to ~27% (13, 14), and these patients have a higher in-hospital mortality compared to patients with at least one SMuRF (14).

To date, there is a paucity of studies that examine the outcomes of this pragmatically challenging group of SMuRF-less patients who present with AMI. They are often overlooked in large clinical trials which rarely report the absence of SMuRFs and are less often recruited into trials targeting atherosclerotic cardiovascular risk intervention. Despite the increasing focus on this group of patients in the west (14–17), SMuRF-less cohort has not been described in Asia. In addition, most previous studies are only limited to the subgroup of SMuRF-less patients presenting with STEMI, with only few studies on patients with non-STEMI (NSTEMI) (15, 18, 19). This study will be the first to describe the prevalence, characteristics, and outcomes of SMuRF-less patients in comparison with those with SMuRF in a large diverse Asian population presenting with AMI.

## Methods

### Setting and Design

Consecutive patients presenting with AMI to a major tertiary academic percutaneous coronary intervention (PCI)-capable hospital in Singapore between January 1, 2011 and March 31, 2021 were retrospectively studied. The hospital is part of the western network that provides PCI services, which include round-the-clock primary PCI, to the western region in Singapore (20). This western network is a hub-and-spoke system that consists of our hospital and two other spoke hospitals. The patients presented with either STEMI or NSTEMI *via* the Emergency Department at the hub hospital or *via* interhospital transfer from the two spoke hospitals.

Patients included in the study were at least 18 years of age and presented with AMI. Patients with previous AMI, PCI, or coronary artery bypass graft (CABG) were excluded. Those with type 2 myocardial infarction diagnoses, defined as the evidence of myocardial infarction with an imbalance between myocardial oxygen supply and demand unrelated to acute coronary atherothrombosis, were excluded (21). SMuRFs (6, 22) were defined as having at least one of the following cardiovascular risk factors: ex-smoker or current smoker, hypertension, diabetes mellitus, or hypercholesterolemia. Hypertension was defined as blood pressure consistently ≥130 and/or ≥80 mm Hg (23) and includes patients previously diagnosed hypertension, prescribed antihypertensives, or newly diagnosed hypertension during the index admission. Diabetes mellitus was defined as previously diagnosed type 1 or 2 diabetes, prescribed glucose lowering medications, or newly diagnosed diabetes using HbA1c levels during the index admission (24). Hypercholesterolemia was defined as previously diagnosed hypercholesterolemia, prescribed lipid-lowering therapy, or newly diagnosed hypercholesterolemia during index admission.

Patients were allocated to 2 study groups according to their SMuRF status: (1) SMuRF, defined as having 1 or more SMuRFs, or (2) SMuRF-less, defined as the absence of SMuRF.

### Data Collection

Data on baseline demographic and clinical characteristics, SMuRF status, previous medical history, clinical status at presentation, angiographic and procedural characteristics, echocardiographic characteristics, and medications on discharge were retrospectively collected from the electronic clinical records. Information on in-hospital complications was also retrieved. The door-to-balloon time was presented only for patients with STEMI. Left ventricular ejection fraction was measured using the Simpson's biplane method and obtained *via* echocardiogram during the hospital stay.

### Study Outcomes

The primary outcome was 30-day cardiovascular mortality. Secondary outcomes were 30-day all-cause mortality, unplanned cardiac readmission, cardiogenic shock, heart failure, and stroke.

Cardiovascular mortality was defined as any death due to any cardiovascular causes, and all-cause mortality was defined as death due to any or unexplained causes. Cardiogenic shock was defined by the presence of persistent hypotension defined as systolic blood pressure <90 mm Hg or mean arterial pressure 30 mm Hg below the baseline, cardiac index (<1.8 L/min/m^2^ without support or <2.2 L/min/m^2^ with support) with adequate or elevated filling pressures (left ventricular end diastolic pressure >18 mm Hg or right ventricular end diastolic pressure >10 to 15 mm Hg) at the time of hospital presentation (25). Heart failure (Killip class ≥3) was defined clinically based on the development of typical signs and symptoms, with structural and functional cardiac abnormalities at the time of presentation (26). Procedural success was defined as <50% residual stenosis with post-PCI thrombolysis in myocardial infarction (TIMI) flow grade 3 (27).

### Statistical Analysis

Statistical analysis was conducted on STATA 16.1 (StataCorp) and IBM SPSS Statistics 25 (SPSS Inc., Chicago, IL, USA). A *p*-value of ≤ 0.05 was considered statistically significant. To compare between baseline characteristics, clinical presentation, and outcomes of included patients, either chi-squared analysis or Fisher's exact test was used to compare categorical and binary variables. Two-sample *t*-test was used in the analysis of continuous variables. A sensitivity analysis of patients without previous heart failure and stroke was also carried out to compare their outcomes. Cardiovascular mortality was assessed in the Fine-Gray model with hazard ratio (HR) to account for competing risk. The issue of competing risk has been well described by Abdel-Qadir et al. (28) In the analysis of binary outcomes including 30-day myocardial infarction, stroke, cardiogenic shock, heart failure and readmission, a generalized linear regression with a log link, gaussian distribution, and robust variance estimator were used to compute the risk ratios (RRs) (29). The RR was preferred due to the ease of interpretation compared to an odds ratio (30). The covariates in the multivariable model included age, sex, ethnicity, chronic kidney disease, AMI type (STEMI and NSTEMI), cardiac arrest, and the presence of left main coronary and/or left anterior descending coronary artery disease. The covariates included in the model were adjudicated based on the significant variates and prognostically important confounders of AMI in concordance with several established AMI studies (11, 14, 15, 20, 31). The Kaplan–Meier survival curves for cardiovascular mortality were constructed from the date of admission up to 30 days. The survival curves were further stratified according to the sex, the three main Asian ethnicities (Chinese, Malay, and Indian), and the AMI type (STEMI and NSTEMI). Additionally, a Kaplan–Meier survival curve of 10-year all-cause mortality rate was also constructed. The study was approved by the local institutional review committee in accordance with the revised Declaration of Helsinki (NHG Research—DSRB: 2021/00089-AMD0001). As the study involved the retrospective analysis of clinically acquired data, the institutional review board waived the need for patient's written informed consent.

## Results

### Study Cohort Characteristics

**Figure 1:**
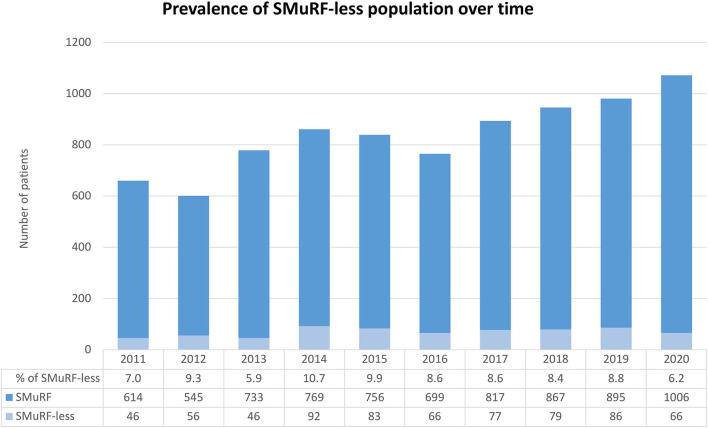
Bar graph displaying the prevalence of the SMuRF-less population from 2011 to 2020.

Of the 8,680 patients with AMI enrolled into the study, 7,934 (91.4%) patients were in the SMuRF group and 746 (8.6%) in the SMuRF-less group (Supplementary Figure 1). The follow-up time was 3.8 (interquartile range [IQR] 1.2–6.7) years and 3.9 (IQR 1.5–7.0) years in SMuRF and SMuRF-less group, respectively. Between 2011 and 2021, the yearly prevalence of SMuRF-less patients presenting with AMI fluctuated little and ranged from 5.9 to 10.7% (Figure 1).

A total of 4,975 (57.3%) patients presented with STEMI and 3,705 (42.7%) with NSTEMI. A larger proportion of SMuRF-less patients presented with STEMI as compared to SMuRF patients (482 [64.6%] vs. 4493 [56.6%], respectively, *p* <0.001). The mean age of SMuRF-less patients was significantly lower than that of SMuRF patients (57 ± 14 vs. 61 ± 13 years, respectively, *p* <0.001). SMuRF-less patients were also less likely to have history of stroke (1.9 vs. 6.0%, respectively, *p* <0.001) and chronic kidney disease (2.3 vs. 10.2%, respectively, *p* <0.001), compared to SMuRF patients. In the SMuRF group, the prevalence of hypertension (60.4%), hypercholesterolemia (58.8%), and diabetes mellitus (41.9%) was high. Among these SMuRF patients, 3,161 (41.2%) were current smokers, 964 (12.6%) ex-smokers, and 3,548 (46.2%) non-smokers. The baseline characteristics of the patients are outlined in Table 1.

**Table 1:** Demographic and clinical characteristics of study cohort presenting with acute myocardial infarction, based on standard modifiable cardiovascular risk factors.

	**Overall (*N* = 8,680)**	**SMuRF (*N* = 7,934)**	**SMuRF-less** **(*N* = 746)**	***P*-Value**
Age	61 (13)	61 (13)	57 (14)	<0.001
BMI	25.3 (4.5)	25.3 (4.5)	25.4 (4.2)	0.623
Male	6,846 (79.1)	6,254 (78.8)	592 (79.4)	0.734
Ethnicity*				<0.001
Chinese	4,889 (61.0)	4,468 (60.9)	421 (62.7)	
Malay	1,697 (21.2)	1,607 (21.9)	90 (13.4)	
Indian	1,367 (17.1)	1,208 (16.5)	159 (23.7)	
Caucasian	56 (0.7)	55 (0.7)	1 (0.1)	
Previous stroke	492 (5.7)	478 (6.0)	14 (1.9)	<0.001
Atrial fibrillation	201 (2.3)	189 (2.4)	12 (1.6)	0.177
Previous heart failure	151 (1.7)	147 (1.9)	4 (0.5)	0.009
Chronic kidney disease	826 (9.5)	809 (10.2)	17 (2.3)	<0.001
Family history of CAD	928 (10.7)	829 (10.5)	99 (13.3)	0.017
Diabetes Mellitus	3,324 (38.3)	3,324 (41.9)	0	<0.001
Hypercholesterolemia	4,663 (53.7)	4,663 (58.8)	0	<0.001
Hypertension	4,793 (55.2)	4,793 (60.4)	0	<0.001
Smoking status*				<0.001
Smoker	3,161 (37.9)	3,161 (41.2)	0	
Ex-smoker	964 (11.5)	964 (12.6)	0	
Non-smoker	4,224 (50.6)	3,548 (46.2)	677 (100.0)	
**Discharge medication**				
ACE-I/ARB	5,454 (62.8)	5,061 (63.8)	393 (52.7)	<0.001
β-blocker	6,804 (78.4)	6,261 (78.9)	543 (72.8)	0.255
Statin	7,855 (90.5)	7,220 (91.0)	635 (85.1)	0.193
Aspirin	7,681 (88.5)	7,053 (88.9)	628 (84.2)	0.679
P2Y12 inhibitor	6,109 (70.4)	5,601 (70.6)	508 (68.1)	0.460
Warfarin	313 (3.6)	280 (3.5)	33 (4.4)	0.150
Direct oral anticoagulants	170 (2.0)	155 (2.0)	15 (2.0)	0.819
Follow-up time (years)	4.4 (3.1)	4.4 (3.1)	4.1 (3.1)	0.030
**Laboratory variables**			
Peak Creatinine (μmol/L)	129 (141)	130 (145)	108 (89)	<0.001
Troponin I (ng/L)	9,465 (18,278)	9,395 (18,232)	10,223 (18,770)	0.251
Left ventricular ejection fraction (%)	49 (13)	49 (13)	49 (13)	0.862
**In-hospital management**				
Presentation route*				0.867
Emergency medical services	2,436 (37.3)	2,215 (37.1)	221 (39.0)	
Walk-in	3,024 (46.3)	2,773 (46.5)	251 (44.3)	
Interhospital transfer	1,034 (15.8)	943 (15.8)	91 (16.0)	
Elective	10 (0.2)	9 (0.2)	1 (0.2)	
Inpatient	27 (0.4)	24 (0.4)	3 (0.5)	
AMI type				<0.001
STEMI	4,975 (57.3)	4,493 (56.6)	482 (64.6)	
NSTEMI	3,705 (42.7)	3,441 (43.4)	264 (35.4)	
Cardiac arrest	252 (2.9)	197 (2.5)	55 (7.4)	<0.001
Underwent PCI	7,429 (92.2)	6,776 (92.5)	653 (90.0)	0.108
Underwent primary PCI	4,641 (93.3)	4,194 (93.3)	447 (92.7)	0.613
Door to balloon time for STEMI patients (min)	48 (36-66)	46 (33-62)	46 (36-61)	0.856
Symptom to balloon time for STEMI patients (min)	202 (129-352)	204 (137-354)	202 (128-351)	0.467
Culprit vessel*				0.002
Left Main	136 (2.0)	116 (1.9)	20 (3.4)	
Left Anterior Descending	3,422 (51.4)	3,086 (50.9)	336 (56.6)	
Circumflex	766 (11.5)	712 (11.8)	54 (9.1)	
Right coronary artery	1,817 (27.3)	1,680 (27.7)	137 (23.1)	
Others	512 (7.7)	465 (7.7)	47 (7.9)	
Number of stents*				0.104
1	3,992 (63.8)	3,610 (63.5)	382 (67.5)	
2	1,219 (19.5)	1,130 (19.9)	89 (15.7)	
≥3	335 (5.4)	307 (5.4)	28 (4.9)	
Number of vessels intervened*				0.582
1	3,972 (88.3)	3,591 (88.4)	381 (87.0)	
2	464 (10.3)	415 (10.2)	49 (11.2)	
≥ 3	63 (1.4)	55 (1.4)	8 (1.8)	
Post-PCI TIMI*				0.352
0	96 (1.6)	84 (1.5)	12 (2.1)	
1	31 (0.5)	26 (0.5)	5 (0.9)	
2	171 (2.8)	156 (2.8)	15 (2.7)	
3	5,859 (95.2)	5,331 (95.2)	528 (94.3)	
Procedural success	6,217 (83.9)	5,658 (83.5)	559 (85.6)	0.231
Coronary artery bypass grafting	225 (2.6)	212 (2.7)	13 (1.7)	0.138

*CAD, Coronary Artery Disease; ACEI, Angiotensin Converting Enzyme Inhibitor; ARB, Angiotensin II Receptor Blocker; AMI, Acute Myocardial Infarction; PCI, Percutaneous Coronary Intervention; STEMI, ST Elevated Myocardial Infarction; NSTEMI, Non-ST Elevated Myocardial Infarction; TIMI, Thrombolysis in Myocardial Infarction.* *Categorical variables are presented as n (%) and continuous variables are presented as Mean (Standard Deviation).* *Non-normally distributed data such as door-to-balloon time and symptom-to-balloon time are reported as median (interquartile range).* *Missing data for ethnicity (n = 665), smoking status (n = 332), presentation route (n = 2,149), culprit vessel (n = 1,880), number of stents (n = 1,172), number of vessels intervened (n = 2,931), post-PCI TIMI (n = 1429).

SMuRF-less patients were more likely to have culprit vessel involving the left anterior descending artery (56.6 vs. 50.9%, respectively) or left main coronary artery (3.4 vs. 1.9%, respectively) compared to SMuRF patients (*p* = 0.002). Both groups of patients did not differ in the rates of overall PCI, primary PCI, symptom-to-door or door-to-balloon time, number of vessels and stents involved in PCI, post-PCI TIMI, PCI success rate, and need for CABG.

SMuRF-less patients also had significantly higher rates of ventricular arrhythmias, and inotropic and invasive ventilation support when compared to SMuRF patients. However, ischemic mitral regurgitation was less common in the SMuRF-less than SMuRF patients. The incidences of other in-hospital complications that include sepsis, atrial fibrillation, bleeding events, and acute kidney injury were similar between the two groups of patients and so was the length of hospital stay (Table 2). On discharge, the SMuRF-less group was less likely to be prescribed ACE inhibitors (ACE-I) or angiotensin-II receptor blockers (ARBs) compared to the SMuRF group.

**Table 2:** In-Hospital complications and outcomes of study cohort presenting with acute myocardial infarction, based on standard modifiable cardiovascular risk factors.

	**Overall (*N* = 8,680)**	**SMuRF (*N* = 7,934)**	**SMuRF-less** **(*N* = 746)**	***P*-Value**
**In-hospital complications**				
Length of hospital stay (days)	6.9 (9.0)	7.0 (9.0)	6.7 (9.0)	0.475
Sepsis	561 (6.5)	520 (6.6)	41 (5.5)	0.265
New onset atrial fibrillation	436 (5.0)	406 (5.1)	30 (4.0)	0.199
Major bleeding	664 (7.6)	613 (7.7)	51 (6.8)	0.397
Inotropic support	832 (9.6)	722 (9.1)	110 (14.7)	<0.001
Intubation	723 (8.3)	626 (7.9)	97 (13.0)	<0.001
Mitral regurgitation	820 (9.4)	768 (9.7)	52 (7.0)	0.018
**Ventricular arrhythmia**				0.009
Nonsustained ventricular tachycardia	413 (4.8)	370 (4.7)	43 (5.8)	
Sustained ventricular tachycardia	188 (2.2)	165 (2.1)	23 (3.1)	
**Acute kidney injury**				0.183
Not requiring dialysis	828 (9.5)	774 (9.8)	54 (7.2)	
Requiring dialysis	129 (1.5)	122 (1.5)	7 (0.9)	
**Outcomes (30-days)**				
All-cause mortality	633 (7.3)	546 (5.5)	87 (11.7)	<0.001
Cardiac related mortality	564 (6.5)	484 (6.1)	80 (10.7)	<0.001
Cardiogenic shock	669 (7.7)	574 (7.2)	95 (12.7)	<0.001
Stroke	167 (1.9)	150 (1.9)	17 (2.3)	0.396
Heart failure	992 (11.4)	924 (11.6)	68 (9.1)	0.117
Readmission	1,142 (13.2)	1,047 (13.2)	95 (12.7)	0.629

*Categorical variables are presented as n (%) and continuous variables are presented as Mean (Standard Deviation)*.

### Study Outcomes

The cardiovascular mortality (10.7 vs. 6.1%, respectively, *p* <0.001), all-cause mortality (11.7 vs. 5.5%, respectively, *p* <0.001), and cardiogenic shock (12.7 vs. 7.2%, respectively, *p* <0.001) were significantly higher in SMuRF-less patients compared to SMuRF patients. The incidences of stroke, hospital readmission, and heart failure were similar between both study groups (Table 2). Sensitivity analysis of patients without prior heart failure or stroke demonstrated similar findings with significantly higher cardiovascular mortality (10.6 vs. 5.4%, respectively, *p* <0.001), all-cause mortality (11.4 vs. 6.1%, respectively, *p* <0.001), and cardiogenic shock (12.9 vs. 7.1%, respectively, *p* <0.001) in SMuRF-less patients compared to SMuRF patients (Supplementary Table 1). An additional sensitivity analysis of patients without prior heart failure, stroke, or chronic kidney disease revealed a similar trend in all-cause mortality, cardiac-related mortality, and cardiogenic shock (Supplementary Table 2).

The Kaplan–Meier curves of 30-day cardiovascular mortality are presented based on study cohort, sex, AMI type, and ethnicity (Supplementary Figure 2). For the overall study cohort, the cumulative event curves diverged early from the day of AMI presentation which indicates higher early mortality in the SMuRF-less group compared to the SMuRF group which was sustained over the 30-day follow-up period (HR 1.837, 95% CI: 1.450–2.328, *p* <0.001). Similar trend was found in the men (HR 2.043, 95% CI: 1.557–2.680, *p* <0.001), but not for women (HR 1.390, 95% CI: 0.852–2.270, *p* = 0.187). For each of the ethnicities, such trend was observed in the Chinese (HR 1.672, 95% CI: 1.222–2.289, *p* = 0.001), Malay (HR 2.904, 95% CI: 1.656–5.094, *p* <0.001), and Indian (HR 2.055, 95% CI: 1.185–3.563, *p* = 0.010) patients. This trend was also observed in the patients with STEMI (HR 1.947, 95% CI: 1.50 −2.527, *p* <0.001) but not in patients with NSTEMI (HR 1.188, 95% CI: 0.658–2.145, *p* = 0.567).

The Kaplan–Meier curves of all-cause mortality over 10 years showed the early separation of mortality rates within the first year of presentation, with higher mortality in the SMuRF-less group (Supplementary Figure 3). However, this mortality difference was attenuated on longer-term follow-up with a crossover of both survival curves at the 5-year mark. Overall, there was no significant difference in all-cause mortality on longer-term follow-up (HR 1.097, 95% CI: 0.909–1.323, *p* = 0.334).

**Figure 2:**
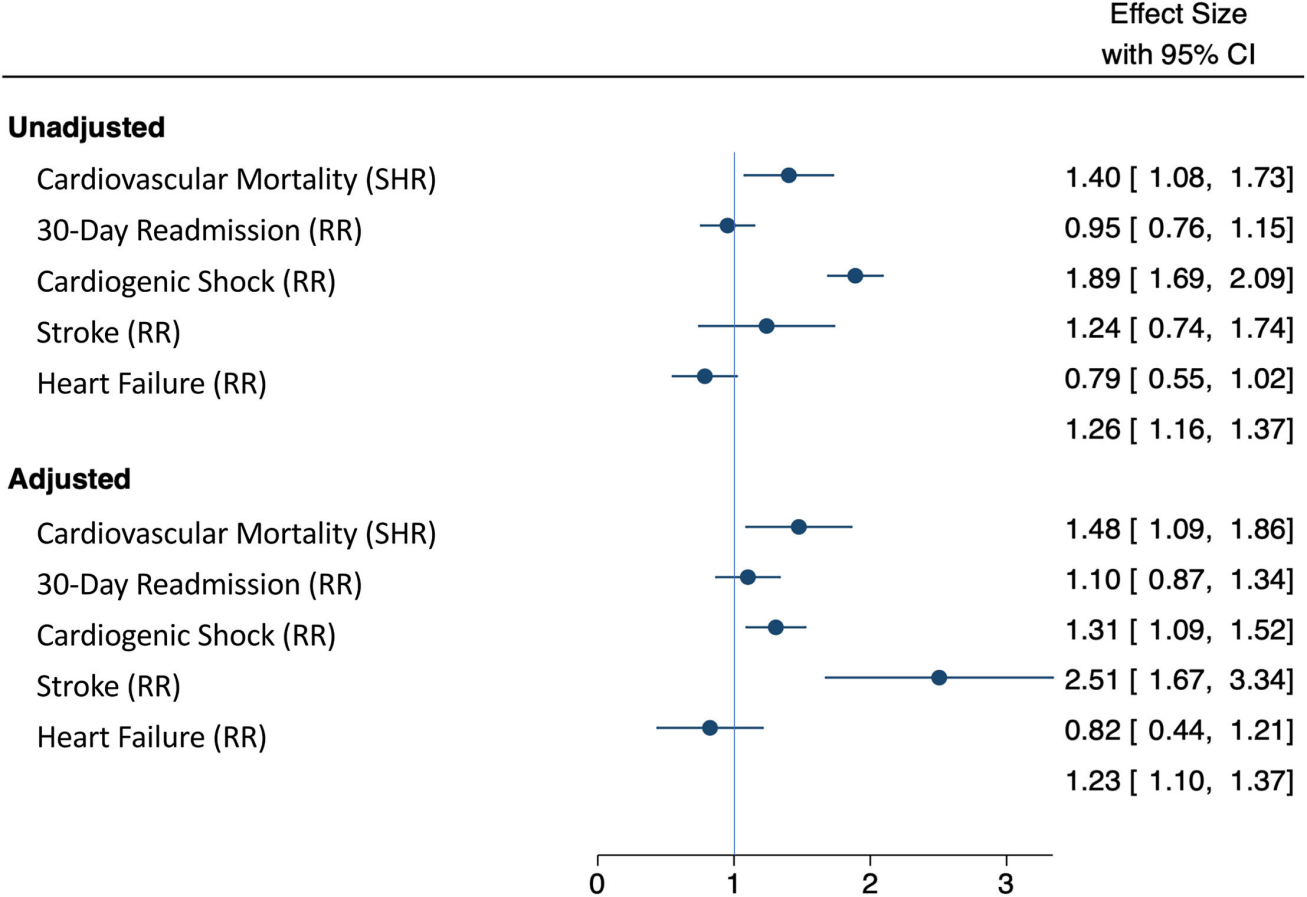
Forest plot comparing unadjusted and adjusted study outcomes. Outcomes were adjusted for age, sex, ethnicity, chronic kidney disease, AMI type (STEMI and NSTEMI), cardiac arrest, and the presence of left main coronary and/or left anterior descending coronary artery disease in SMuRF-less and SMuRF patients presenting with AMI.

The multivariable analysis showed that SMuRF-less patients had higher risk of 30-day cardiovascular mortality (HR 1.48, 95% CI: 1.09–1.86, *p* = 0.048), cardiogenic shock (RR: 1.31, 95% CI: 1.09–1.52, *p* = 0.015), and stroke (RR: 2.51, 95% CI: 1.67–3.34, *p* = 0.030) compared to the SMuRF patients despite adjusting for important confounders (Figure 2). The multivariate analysis also demonstrated that the other factors that influence cardiovascular mortality rates include age, ethnicity, AMI type, chronic renal failure, cardiac arrest, and left main and/or left anterior descending disease (Supplementary Table 3). The risk of unplanned cardiac readmission (RR: 1.10, 95% CI: 0.87–1.39, *p* = 0.413) and heart failure (RR: 0.82, 95% CI: 0.56–1.21, *p* = 0.326) was similar between both patient groups. The competing risk analysis for in-hospital cardiovascular mortality is shown in Supplementary Table 4.

## Discussion

This study is the first to examine the prognostic outcomes of an often overlooked subset of patients without SMuRFs in a typically understudied Asian population presenting with AMI. It is also the first of such study to include both patients with NSTEMI and STEMI. The main findings of the study are as follows: (1) The prevalence of SMuRF-less patients presenting with AMI in an Asian cohort was 8.6%, with its yearly prevalence relatively constant over the past decade; (2) SMuRF-less patients tend to present in a more critical state compared to SMuRF patients, with higher rates of ventricular arrhythmia, and requirement for inotropic and invasive ventilation support; (3) The adjusted risks of cardiovascular mortality, cardiogenic shock, and stroke were significantly higher in the SMuRF-less patients compared to SMuRF patients; (4) The significantly higher cardiovascular mortality in SMuRF-less patients compared to SMuRF patients was apparent early from presentation and was sustained over 30 days. Such trend was observed in men and patients with STEMI, but not in women or patients with NSTEMI. Similar trend was also seen across all three Asian ethnicities. This significant difference in mortality was attenuated over time, with a crossover in survival curves around the 5-year mark.

Traditionally, both primary and secondary prevention of cardiovascular diseases have been focused on high-risk individuals with cardiovascular risk factors (18, 32). As a result, the subgroup of patients without SMuRFs remains understudied. The recent studies on both STEMI and NSTEMI have demonstrated an increasing prevalence of patients without traditional risk factors (13), with the prevalence of SMuRF-less patients being 10.5% in the USA (19), 14.5% in Canada (15), 14.9% in Sweden (11), and 19–25% in Australia (13, 14). Notably, the population of SMuRF-less patients in our Asian cohort was much lower with the prevalence of only 8.6%. The stark difference in the proportion of SMuRF-less patients across the globe might be partly explained by the differences in risk factor identification (33), genetic predisposition (34), lifestyle factors such as smoking and physical activity (35, 36), and individual country's primary prevention program (33, 37). Even with the presence of traditional cardiovascular risk factors, their impacts might vary across different ethnic groups, with stroke being more common among hypertensive patients in Asia and chronic heart disease more prevalent in the west (38). Despite relatively lower than that seen in the west, the prevalence of SMuRF-less patients in our Asian cohort remains sizeable and warrants further attention to address specific modifiable factors that might predispose Asians to various cardiovascular comorbidities.

Even though the SMuRF-less patients in our cohort were generally younger and had fewer baseline comorbidities, their cardiovascular mortality was higher than those with conventional risk factors. This is consistent with the findings from previous studies (11, 14–16, 19) based in the west and could be partly explained by multiple postulated reasons. Several cardiovascular risk factors, such as serum cholesterol or glycated hemoglobin A1c, have a linear relationship with the risk of cardiovascular morbidity, and categorizing the patients into binary groups using a standard diagnostic threshold can potentially introduce selection bias by missing out on patients with borderline measurements for certain risk factors that have not reached the diagnostic thresholds. As mentioned earlier, individuals with pre-disease state for various cardiovascular risk factors might also have a higher atherosclerotic cardiovascular risk. Moreover, the role of less well established risk factors such as body mass index, triglyceride concentrations, high-density lipoprotein concentration, and sedentary lifestyle might also be the potential drivers of atherosclerosis, but have not been concomitantly evaluated. Additionally, some recognized risk factors such as abdominal obesity, psychosocial factors, sedentary lifestyle, dietary factors, and alcohol consumption are not easily quantified, and hence, their potential impact on the outcome of SMuRF-less patients is not well assessed (39). Furthermore, the use of aspirin, statins, and ACE-I/ARBs has been associated with reduced cardiovascular events, mortality, and STEMI presentations (40–42). As patients with known risk factors are more likely to be on such treatment, the AMI severity may have been modified by evidence-based primary prevention therapy (16), which leads to better outcomes among the SMuRF patients. We as well as Figtree et al. reported higher rates of left main and left anterior descending culprit in SMuRF-less patients compared to those with risk factors (11). Although this may partly be explained by the family history of premature coronary artery disease, this is an important finding as it contributes to an adverse AMI risk profile in SMuRF-less patients. There were also higher STEMI presentations among SMuRF-less patients compared to their counterparts, while increased NSTEMI presentations were observed among those with SMuRFs as opposed to those without SMuRFs. This is indeed hypothesis-generating as it sheds light on the postulated reasons underlying the adverse, and distinct, risk profile of SMuRF-less individuals. The emerging evidence calls for larger international efforts in identifying novel mechanisms that contribute to host susceptibility in developing atherosclerosis and thrombosis despite the absence of cardiovascular risk factors.

The pathogenesis of atherosclerosis, especially its genetic basis, is also not fully understood. A recent study reported as many as 55 genetic loci that are associated with coronary artery disease, with more than 66% of them not linked to the traditional risk factors (43). Compared to the patients with SMuRF, more SMuRF-less patients in our study were of Indian ethnicity and had family history of premature coronary artery disease. This is highly suggestive of a genetic predilection in SMuRF-less patients to develop atherosclerotic cardiovascular disease. In addition, the SMURF-less patients in our cohort were 4 years younger on average than patients with SMURFs, which differs from the SWEDEHEART cohort of patients with STEMI (11), in which SMuRF-less patients were older. This might suggest that there is a stronger role of genetic factors in an Asian population, which results in earlier AMI presentation of SMuRF-less patients. It is plausible that these genetic factors might play a major role in the disease process among SMuRF-less patients, which leads to the onset of disease at a younger age and more advanced disease at presentation with consequent worse prognosis.

Our study found an increased short-term cardiovascular mortality only in the male SMuRF-less patients, but not in female SMuRF-less patients. The reasons underlying sex differences in short-term mortality are complex and largely dependent on age and AMI type. Several studies have shown that the higher 30-day mortality post-AMI in women, compared to men, is most pronounced in young- and middle-aged individuals (44), with this sex difference diminishing after the age of 60 (45). As such, the likely possibility that no significant increase in short-term mortality was observed in SMuRF-less women was due to the older age profile (mean 64 ± 4 years) at the point of AMI and with fewer STEMI presentations (50%), compared to the SMuRF-less men who presented younger (mean 54 ± 12 years) with more STEMI presentations (68.4%).

Moreover, significant mortality difference between our SMuRF and SMuRF-less patients was only observed in the STEMI, but not patients with NSTEMI. This is in contrast to a prior study that showed increased mortality in SMuRF-less as compared to SMuRF patients with NSTEMI (19). One possible reason for this discrepancy is the significantly lower mortality events in NSTEMI as compared to patients with STEMI (4.1 vs. 8.3% respectively, *p* <0.001), which might lead to less apparent difference seen between SMuRF and SMuRF-less patients among our patients with NSTEMI (46). Although patients with type 2 myocardial infarction were excluded from the study, patients with NSTEMI remain a heterogenous group with respect to their risk profiles. Certain risk factors such as diabetes or current smoking have been demonstrated to disproportionally increase the risk of obstructive coronary artery disease in women, and women with obstructive coronary artery disease had the higher 30-day mortality than men (47). In our study, women tended to present with NSTEMI (57.9%) than STEMI (42.1%). On the contrary, men tended to present with STEMI (61.4%) than NSTEMI (38.6%). The recent evidence has suggested significant increase in mortality among women compared to men persisted till the age > 85 in the STEMI cohort, but only persisted till the age <65 in the NSTEMI cohort (44). Currently, there is a paucity of studies focused on the SMuRF-less patients in NSTEMI. Hence, further studies with a larger study cohort will be the next important step to better understand the difference in outcomes in patients with NSTEMI.

We found that Indians made up 17.1% of patients with AMI, although they only constitute 7.5% of the Singapore population, as opposed to 76.0% being Chinese, 15.0% Malay, and 1.5% other ethnicities (48). Previous cohort studies have found that Indians have a higher risk of developing ACS (49–51). This could be accounted for by the variation in the demographic, socioeconomic, and health characteristics of each ethnic group (52). Notably, a significant portion of Indian patients presented as SMuRF-less, which could indicate underlying genetic factors predisposing them to AMI. This is hypothesis generating as it reflects the large interethnic variation within the Asian cohort, and calls for further prospective studies to explore the pathomechanisms underlying the ethnic disparity in SMuRF-less patients presenting with AMI.

For the first time, long-term survival outcomes of SMuRF-less patients with AMI in an Asian population have been detailed. The all-cause mortality remained higher in the SMuRF-less group for slightly over 5 years in patients with AMI. The early separation of the cumulative mortality event curves was observed, with a gradual reduction in the curve separation after 30 days. The differences in all-cause mortality occurred primarily early on, during in-hospital stay, with parallel event rates thereafter to 30 days. This is concordant with the findings from Figtree et al. (11), where the survival curves crossed at the 8th year for men and the 12th year for women. The early separation of the curves could possibly be attributed to higher rates of arrhythmia found in the SMuRF-less cohort, which is a common cause of early mortality in patients post-myocardial infarction (53). Subsequently, the SMuRF-less patients who survived through the early stages of AMI had improved survival over the long-term follow-up in relation to the SMuRF patients.

Similar to the current literature (11, 15, 16), we found that SMuRF-less patients were less likely to be treated with guideline-directed medication including ACE-I or ARBs when compared to the SMuRF patients. Figtree et al. (11) have shown that suboptimal prescription rate of ACE-I or ARBs and beta-blockers was directly correlated with a higher mortality among the SMuRF-less patients which is in line with other studies that demonstrate the prognostic benefit of early initiation of beta-blocker and ACE-I in patients with AMI (54–56). The reason that the SMuRF-less patients were less likely to be prescribed with prognostically important medication was unclear but could be related to the false perception that they were of lower cardiac risk. The worse clinical status at presentation, the lack of preexisting hypertension, and higher incidence of stroke among the SMuRF-less patients might lead to a poorer hemodynamics which precluded the use of beta-blockers or ACE-I or ARBs. Increased awareness of the paradoxical unfavorable outcome in SMuRF-less patients presenting with AMI should be widely promoted, and early initiation of guideline-directed medical therapy among AMI patients remains crucial regardless of the cardiovascular risk factor status.

### Clinical Implications

Our findings raise concerns regarding the unfavorable outcome in SMuRF-less patients presenting with AMI among the Asian population. Such patients are not uncommon and may present in an even worse clinical state than those with one or more standard cardiovascular risk factors. These ameliorate the general sense of complacency that significant coronary artery disease is an unlikely health concern in individuals without cardiovascular risk factors. Clinicians need to be aware of this unexplained paradoxical phenomenon, and effective lifestyle and pharmacological intervention need to be optimized in all patients regardless of their SMuRF status. Although lower than that reported in the west, the proportion of SMuRF-less patients in our Asian population remain sizeable, which indicates that this is a global phenomenon that warrants its due attention by all health-care systems. More efforts are needed to understand the underlying pathophysiology of atherosclerotic cardiovascular risk factors in SMuRF-less patients, from the onset of atherosclerosis through its progression and the occurrence of AMI, in order to identify such individuals, so that appropriate and timely preventative intervention can be given. Currently, most published studies were limited to short-term outcomes, and hence, further studies are also needed in order to understand the long-term outcome of SMuRF-less patients with AMI.

### Strengths and Limitations

This study is the first to examine the prognosis of SMuRF-less patients presenting with AMI in a large Asian cohort. However, this study has its limitations. First, this is a single-center retrospective observational study, which might be affected by unknown confounders and bias. Therefore, causality cannot be deduced from our results. Nevertheless, this study offers a large sample of real-world data on the outcomes of consecutive SMuRF-less patients presenting with AMI. In addition, such potential bias was mitigated by adjusting for important covariates in the multivariable models and using mortality as the primary study outcome. Second, the current method of categorizing patients into SMuRF and SMuRF-less groups might not be ideal, but it is the universal method used by all published studies, and based on the local or international diagnostic threshold for each of the SMuRFs. Such thresholds are generally derived based on the clinical evidence or expert consensus and usually form the thresholds for guideline-directed treatment. Third, some recognized atherosclerotic cardiovascular risk factors other than those universally considered as SMuRF are also beyond the scope for the evaluation in this study. The retrospective nature of the study did not allow further evaluation of SMuRF-less patients for non-atherosclerotic cause of AMI such as proteins C and S deficiency.

## Conclusion

Patients presenting with AMI but without any SMuRF are not uncommon in a multiethnic Asian population. They tend to present in a worse clinical state and have poorer short-term outcomes that include higher cardiovascular mortality, compared to those with SMuRF.

## Data Availability Statement

The raw data supporting the conclusions of this article will be made available by the authors, without undue reservation.

## Ethics Statement

The studies involving human participants were reviewed and approved by NHG Research—DSRB: 2021/00089-AMD0001. Written informed consent for participation was not required for this study in accordance with the national legislation and the institutional requirements.

## Author Contributions

GK and NC: conceptualization, methodology, formal analysis, investigation, data curation, writing—original draft, writing, reviewing, and editing. CN and YC: writing—original draft, writing, reviewing, and editing. OL, AA, GN, WK, K-KP, RF, JY, T-CY, AL, C-HL, MC, H-CT, and P-HL: writing, reviewing, editing, and supervision. All authors contributed to the article and approved the submitted version.

## Conflict of Interest

The authors declare that the research was conducted in the absence of any commercial or financial relationships that could be construed as a potential conflict of interest.

## Publisher's Note

All claims expressed in this article are solely those of the authors and do not necessarily represent those of their affiliated organizations, or those of the publisher, the editors and the reviewers. Any product that may be evaluated in this article, or claim that may be made by its manufacturer, is not guaranteed or endorsed by the publisher.

## Supplementary Material

Supplementary Figure 1 Flow chart of study population.

Supplementary Figure 2 Kaplan–Meier curves for the 30-day cardiovascular mortality in all patients presenting with AMI, with subgroup analysis based on sex, ethnicity, and AMI type.

Supplementary Figure 3 Kaplan–Meier curve for long-term all-cause mortality in all patients presenting with AMI.

Supplementary Table 1 Outcomes of SMuRF-less compared to SMuRF patients, in patients without previous heart failure or stroke.

Supplementary Table 2 Outcomes of SMuRF-less compared to SMuRF patients, in patients without previous heart failure, stroke, or chronic kidney disease.

Supplementary Table 3 Multivariate analysis of the 30-day cardiovascular mortality between SMuRF and SMuRF-less.

Supplementary Table 4 In-hospital cardiovascular mortality assessed in the Fine-Gray model to account for competing risk.

## References

[1] Giampaoli S Palmieri L Mattiello A Panico S. Definition of high risk individuals to optimise strategies for primary prevention of cardiovascular diseases. Nutri Metabol Cardiovascul Dis. (2005) 15:79–85. 10.1016/j.numecd.2004.12.001 15871855

[2] Verschuren WM Jacobs DR Bloemberg BP Kromhout D Menotti A Aravanis C. Serum total cholesterol and long-term coronary heart disease mortality in different cultures: twenty-five—year follow-up of the seven countries study. JAMA. (1995) 274:131–6. 10.1001/jama.274.2.131 7596000

[3] MacMahon S Peto R Collins R Godwin J Cutler J Sorlie P. Blood pressure, stroke, and coronary heart disease: part 1, prolonged differences in blood pressure: prospective observational studies corrected for the regression dilution bias. The Lancet. (1990) 335:765–74. 10.1016/0140-6736(90)90878-9 1969518

[4] Health UDo Services H. The Health Benefits of Smoking Cessation: a report of the surgeon general dhhs publication no.(cdc) 90-8416. Rockville, Maryland: US Department of Health and Human Services. Public Health Service, Centers for Disease Control, Center for Chronic Disease Prevention and Health Promotion, Office on Smoking and Health. (1990).

[5] Stamler J Vaccaro O Neaton JD Wentworth D Group MRFITR. Diabetes, other risk factors, and 12-yr cardiovascular mortality for men screened in the multiple risk factor intervention trial. Diabetes Care. (1993) 16:434–44. 10.2337/diacare.16.2.434 8432214

[6] TR D FE M GV M. Coronary heart disease in the Framingham study. Am J Public Health Nation's Health. (1957) 47:4–24. 10.2105/AJPH.47.4_Pt_2.4 13411327 PMC1550985

[7] Kannel WB Dawber TR Kagan A Revotskie N STOKES III J. Factors of risk in the development of coronary heart disease—six-year follow-up experience: the Framingham Study. Ann Intern Med. (1961) 55:33–50. 10.7326/0003-4819-55-1-33 13751193

[8] Wilson PW D'Agostino RB Levy D Belanger AM Silbershatz H Kannel WB. Prediction of coronary heart disease using risk factor categories. Circulation. (1998) 97:1837–47. 10.1161/01.CIR.97.18.1837 9603539

[9] Ridker PM Buring JE Rifai N Cook NR. Development and validation of improved algorithms for the assessment of global cardiovascular risk in women: the Reynolds Risk Score. JAMA. (2007) 297:611–9. 10.1001/jama.297.6.611 17299196

[10] Emberson J Whincup P Morris R Walker M Ebrahim S. Evaluating the impact of population and high-risk strategies for the primary prevention of cardiovascular disease. Euro Heart J. (2004) 25:484–91. 10.1016/j.ehj.2003.11.012 15039128

[11] Figtree GA Vernon ST Hadziosmanovic N Sundström J Alfredsson J Arnott C. Mortality in STEMI patients without standard modifiable risk factors: a sex-disaggregated analysis of SWEDEHEART registry data. The Lancet. (2021) 397:1085–94. 10.1016/S0140-6736(21)00272-5 33711294

[12] Piepoli MF Hoes AW Agewall S Albus C Brotons C Catapano AL. Guidelines: Editor's choice: (2016). European Guidelines on cardiovascular disease prevention in clinical practice: The Sixth Joint Task Force of the European Society of Cardiology and Other Societies on Cardiovascular Disease Prevention in Clinical Practice (constituted by representatives of 10 societies and by invited experts) Developed with the special contribution of the European Association for Cardiovascular Prevention & Rehabilitation (EACPR). Euro Heart J. (2016) 37:2315. 10.1093/eurheartj/ehw106 27222591 PMC4986030

[13] Vernon ST Coffey S Bhindi R Soo Hoo SY Nelson GI Ward MR. Increasing proportion of ST elevation myocardial infarction patients with coronary atherosclerosis poorly explained by standard modifiable risk factors. Euro J Prevent Cardiol. (2017) 24:1824–30. 10.1177/2047487317720287 28703626

[14] Vernon ST Coffey S D'Souza M Chow CK Kilian J Hyun K. ST-Segment–Elevation Myocardial Infarction (STEMI) patients without standard modifiable cardiovascular risk factors—how common are they, and what are their outcomes? J Am Heart Assoc. (2019) 8:e013296. 10.1161/JAHA.119.013296 31672080 PMC6898813

[15] Wang JY Goodman SG Saltzman I Wong GC Huynh T Dery JP. Cardiovascular risk factors and in-hospital mortality in acute coronary syndromes: insights from the canadian global registry of acute coronary events. Can J Cardiol. (2015) 31:1455–61. 10.1016/j.cjca.2015.04.007 26143140

[16] Canto JG Kiefe CI Rogers WJ Peterson ED Frederick PD French WJ. Number of coronary heart disease risk factors and mortality in patients with first myocardial infarction. Jama. (2011) 306:2120–7. 10.1001/jama.2011.1654 22089719 PMC4494683

[17] Jernberg T Attebring MF Hambraeus K Ivert T James S Jeppsson A. The Swedish Web-system for enhancement and development of evidence-based care in heart disease evaluated according to recommended therapies (SWEDEHEART). Heart. (2010) 96:1617–21. 10.1136/hrt.2010.198804 20801780

[18] Khot UN Khot MB Bajzer CT Sapp SK Ohman EM Brener SJ. Prevalence of conventional risk factors in patients with coronary heart disease. Jama. (2003) 290:898–904. 10.1001/jama.290.7.898 12928466

[19] Roe MT Halabi AR Mehta RH Chen AY Newby LK Harrington RA. Documented traditional cardiovascular risk factors and mortality in non–ST-segment elevation myocardial infarction. Am Heart J. (2007) 153:507–14. 10.1016/j.ahj.2006.12.018 17383286

[20] Chew NW Sia C-H Wee H-L Jia-Da Benedict L Rastogi S Kojodjojo P. Impact of the COVID-19 pandemic on door-to-balloon time for primary percutaneous coronary intervention—results from the singapore western STEMI network. Circulat J. (2021) 85:139–49. 10.1253/circj.CJ-20-0800 33162491

[21] Thygesen K Alpert JS Jaffe AS Chaitman BR Bax JJ Morrow DA. Fourth universal definition of myocardial infarction (2018). European heart journal. (2019) 40:237–69. 10.1093/eurheartj/ehy856 30165617

[22] Piironen M Ukkola O Huikuri H Havulinna AS Koukkunen H Mustonen J. Trends in long-term prognosis after acute coronary syndrome. Euro J Prevent Cardiol. (2017) 24:274–80. 10.1177/2047487316679522 27856805

[23] Whelton PK Carey RM Aronow WS Casey DE Collins KJ Dennison Himmelfarb C. ACC/AHA/AAPA/ABC/ACPM/AGS/APhA/ASH/ASPC/NMA/PCNA guideline for the prevention, detection, evaluation, and management of high blood pressure in adults: a report of the American college of cardiology/american heart association task force on clinical practice guidelines. J Am Coll Cardiol. (2018) 71:e127–248. 10.1161/HYP.0000000000000076 29146535

[24] Deedwania P Kosiborod M Barrett E Ceriello A Isley W Mazzone T. Hyperglycemia and acute coronary syndrome: a scientific statement from the American heart association diabetes committee of the council on nutrition, physical activity, and metabolism. Circulation. (2008) 117:1610–9. 10.1161/CIRCULATIONAHA.107.188629 18299505

[25] Hochman JS Sleeper LA Godfrey E McKinlay SM Sanborn T Col J. Should we emergently revascularize occluded coronaries for cardiogenic shock: an international randomized trial of emergency PTCA/CABG—trial design. Am Heart J. (1999) 137:313–21. 10.1053/hj.1999.v137.95352 9924166

[26] Ponikowski P Voors AA Anker SD Bueno H Cleland JG Coats AJ. ESC Guidelines for the diagnosis and treatment of acute and chronic heart failure: The Task Force for the diagnosis and treatment of acute and chronic heart failure of the European Society of Cardiology (ESC) Developed with the special contribution of the Heart Failure Association (HFA) of the ESC. Euro Heart J. (2016) 37:2129–200. 10.1093/eurheartj/ehw128 27206819

[27] George S Cockburn J Clayton TC Ludman P Cotton J Spratt J. Long-term follow-up of elective chronic total coronary occlusion angioplasty: analysis from the UK central cardiac audit database. J Am Coll Cardiol. (2014) 64:235–43. 10.1016/j.jacc.2014.04.040 25034057

[28] Abdel-Qadir H Fang J Lee DS Tu JV Amir E Austin PC. Importance of considering competing risks in time-to-event analyses: application to stroke risk in a retrospective cohort study of elderly patients with atrial fibrillation. Circul Cardiovascul Qual Outcomes. (2018) 11:e004580. 10.1161/CIRCOUTCOMES.118.004580 29997149 PMC7665273

[29] Cummings P. Methods for estimating adjusted risk ratios. The Stata J. (2009) 9:175–96. 10.1177/1536867X0900900201

[30] Cummings P. The relative merits of risk ratios and odds ratios. Archiv Pediatr Adolesc Med. (2009) 163:438–45. 10.1001/archpediatrics.2009.31 19414690

[31] Phua K Chew NW Sim V Zhang AA Rastogi S Kojodjojo P. One-year outcomes of patients with ST-segment elevation myocardial infarction during the COVID-19 pandemic. J Thrombosis Thrombol. (2021) 2021:1-11. 10.1007/s11239-021-02557-6 PMC8390088 34448103

[32] Manuel DG Lim J Tanuseputro P Anderson GM Alter DA Laupacis A. Revisiting Rose: strategies for reducing coronary heart disease. Bmj. (2006) 332:659–62. 10.1136/bmj.332.7542.659 16543339 PMC1403258

[33] Perkovic V Huxley R Wu Y Prabhakaran D MacMahon S. The burden of blood pressure-related disease: a neglected priority for global health. Hypertension. (2007) 50:991–7. 10.1161/HYPERTENSIONAHA.107.095497 17954719

[34] Cullen P Funke H Schulte H Assmann G. Lipoproteins and cardiovascular risk-from genetics to CHD prevention. Journal of atherosclerosis and thrombosis. (1997) 4:51–8. 10.5551/jat1994.4.51 9638514

[35] Teo K Lear S Islam S Mony P Dehghan M Li W. Prevalence of a healthy lifestyle among individuals with cardiovascular disease in high-, middle-and low-income countries: the prospective urban rural epidemiology (PURE) study. Jama. (2013) 309:1613–21. 10.1001/jama.2013.3519 23592106

[36] Ueshima H Sekikawa A Miura K Turin TC Takashima N Kita Y. Cardiovascular disease and risk factors in Asia: a selected review. Circulation. (2008) 118:2702–9. 10.1161/CIRCULATIONAHA.108.790048 19106393 PMC3096564

[37] Mendis S Abegunde D Yusuf S Ebrahim S Shaper G Ghannem H. WHO study on Prevention of REcurrences of Myocardial Infarction and StrokE (WHO-PREMISE). Bull World Health Organiz. (2005) 83:820–9. 16302038 PMC2626468

[38] Liu LS. [2010 Chinese guidelines for the management of hypertension]. Zhonghua Xin Xue Guan Bing Za Zhi. (2011) 39:579−615. 22088239

[39] Yusuf S Hawken S Ôunpuu S Dans T Avezum A Lanas F. Effect of potentially modifiable risk factors associated with myocardial infarction in 52 countries (the INTERHEART study): case-control study. lancet. (2004) 364:937–52. 10.1016/S0140-6736(04)17018-9 15364185

[40] Bergami M Cenko E Yoon J Mendieta G Kedev S Zdravkovic M. Statins for primary prevention among elderly men and women. Cardiovascul Res. (2021) 21:348. 10.1093/cvr/cvab348 34864917 PMC9648819

[41] Bugiardini R Pavasović S Yoon J van der Schaar M Kedev S Vavlukis M. Aspirin for primary prevention of ST segment elevation myocardial infarction in persons with diabetes and multiple risk factors. EClinicalMed. (2020) 27:100548. 10.1016/j.eclinm.2020.100548 33150322 PMC7599315

[42] Bugiardini R Yoon J Kedev S Stankovic G Vasiljevic Z Miličić D. Prior beta-blocker therapy for hypertension and sex-based differences in heart failure among patients with incident coronary heart disease. Hypertension. (2020) 76:819–26. 10.1161/HYPERTENSIONAHA.120.15323 32654558

[43] Deloukas P Kanoni S Willenborg C Farrall M Assimes TL Thompson JR. Large-scale association analysis identifies new risk loci for coronary artery disease. Nature genetics. (2013) 45:25–33. 10.1038/ng.2480 23202125 PMC3679547

[44] Alkhouli M Alqahtani F Jneid H Al Hajji M Boubas W Lerman Aors. Age-stratified sex-related differences in the incidence, management, and outcomes of acute myocardial infarction. Mayo Clinic Proceedings. London: Elsevier (2021). 10.1016/j.mayocp.2020.04.048 33483147

[45] Cenko E Yoon J Kedev S Stankovic G Vasiljevic Z Krljanac G. Sex differences in outcomes after STEMI: effect modification by treatment strategy and age. JAMA Intern Med. (2018) 178:632–9. 10.1001/jamainternmed.2018.0514 29630703 PMC6145795

[46] Cecchi E D'Alfonso MG Chiostri M Parigi E Landi D Valente S. Impact of hypertension history on short and long-term prognosis in patients with acute myocardial infarction treated with percutaneous angioplasty: comparison between STEMI and NSTEMI. High Blood Pressure Cardiovascul Prevent. (2014) 21:37–43. 10.1007/s40292-013-0032-1 24218158

[47] Manfrini O Yoon J van der Schaar M Kedev S Vavlukis M Stankovic G. Sex differences in modifiable risk factors and severity of coronary artery disease. J Am Heart Assoc. (2020) 9:e017235. 10.1161/JAHA.120.017235 32981423 PMC7792418

[48] gov.sg. What are the racial proportions among Singapore citizens? (2019). [Available from: gov.sg.

[49] Wong CP Loh SY Loh KK Ong PJL Foo D Ho HH. Acute myocardial infarction: Clinical features and outcomes in young adults in Singapore. World J Cardiol. (2012) 4:206. 10.4330/wjc.v4.i6.206 22761974 PMC3386311

[50] Lee J Heng D Chia KS Chew SK Tan BY Hughes K. Risk factors and incident coronary heart disease in Chinese, Malay and Asian indian males: the singapore cardiovascular cohort study. Int J Epidemiol. (2001) 30:983–8. 10.1093/ije/30.5.983 11689508

[51] Mak K-H Chia K-S Kark J Chua T Tan C Foong B-H. Ethnic differences in acute myocardial infarction in Singapore. Euro Heart J. (2003) 24:151–60. 10.1016/S0195-668X(02)00423-2 12573272

[52] Rogers RG Hummer RA Nam CB Peters K. Demographic, socioeconomic, and behavioral factors affecting ethnic mortality by cause. Social Forces. (1996) 74:1419–38. 10.2307/2580357

[53] Solomon SD Zelenkofske S McMurray JJ Finn PV Velazquez E Ertl G. Sudden death in patients with myocardial infarction and left ventricular dysfunction, heart failure, or both. New Engl J Med. (2005) 352:2581–8. 10.1056/NEJMoa043938 15972864

[54] Bugiardini R Cenko E Ricci B Vasiljevic Z Dorobantu M Kedev S. Comparison of early versus delayed oral β blockers in acute coronary syndromes and effect on outcomes. Am J Cardiol. (2016) 117:760–7. 10.1016/j.amjcard.2015.11.059 26778165

[55] Andersson C Shilane D Go AS Chang TI Kazi D Solomon MD. Beta-blocker therapy and cardiac events among patients with newly diagnosed coronary heart disease. J Am Coll Cardiol. (2014) 64:247–52. 10.1016/j.jacc.2014.04.042 25034059

[56] Group I-C. ISIS-4: a randomized factorial trial assessing early oral captopril, oral mononitrate, and intravenous magnesium sulfate in 58,050 patients with suspected acute myocardial infarction. Lancet. (1995) 345:669–85. 10.1016/S0140-6736(95)90865-X 7661937

