# Supplementary figures and images for: Prognostic Outcomes in Acute Myocardial Infarction Patients Without Standard Modifiable Risk Factors: A Multiethnic Study of 8,680 Asian Patients
Source: Front Cardiovasc Med. 2022 Mar 29;9:869168. doi: 10.3389/fcvm.2022.869168 (PMC9001931; doi:10.3389/fcvm.2022.869168)

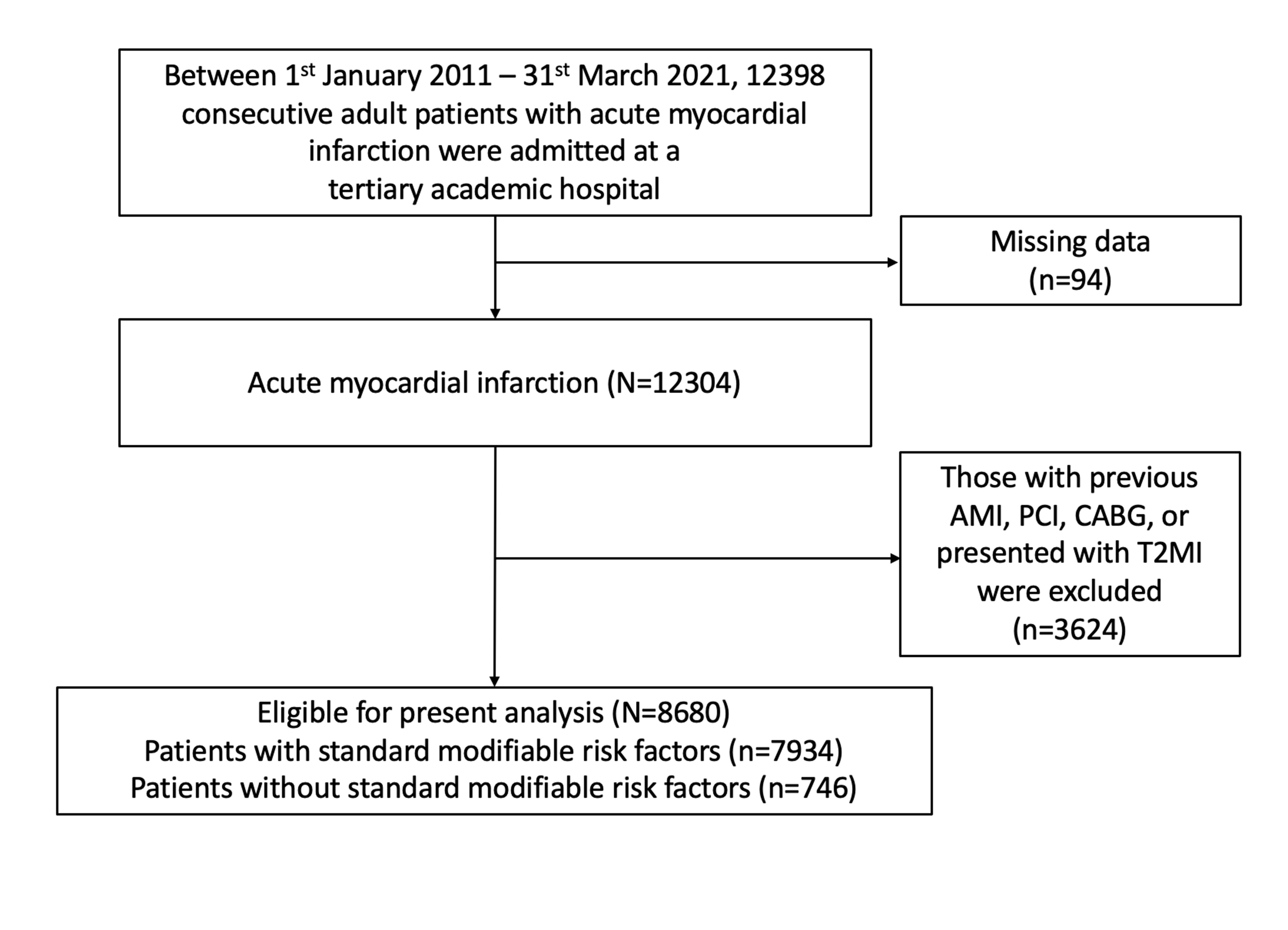

Supplement: Supplementary Figure 1 — Flow chart of study population. [file Image_1.TIFF]

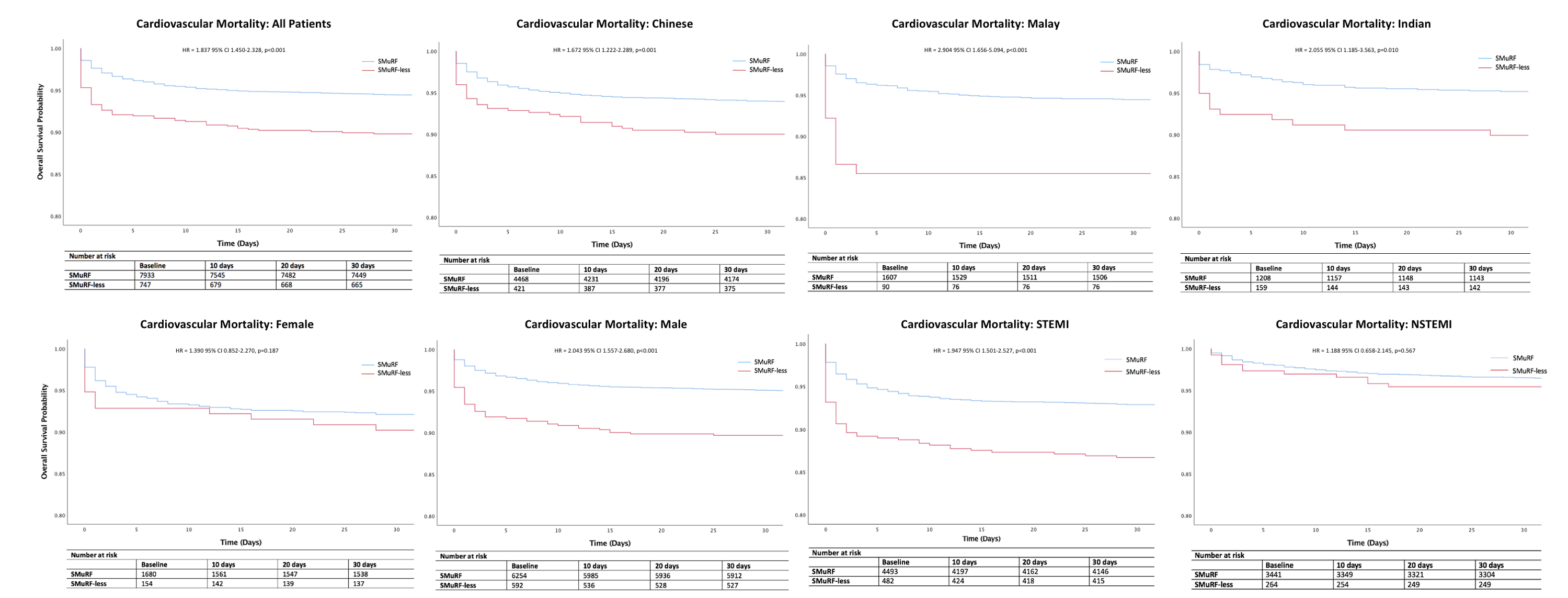

Supplement: Supplementary Figure 2 — Kaplan–Meier curves for the 30-day cardiovascular mortality in all patients presenting with AMI, with subgroup analysis based on sex, ethnicity, and AMI type. [file Image_2.TIFF]

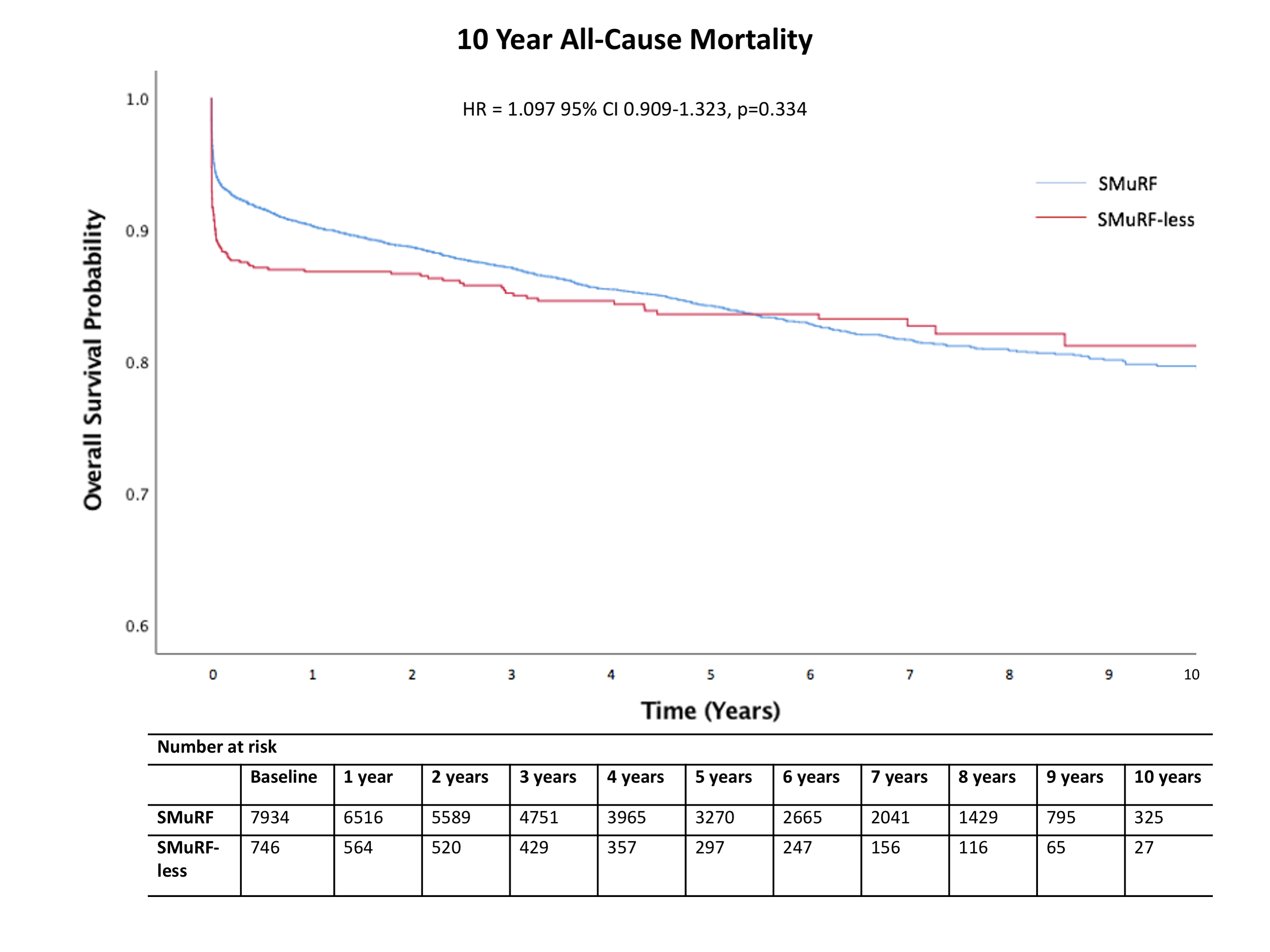

Supplement: Supplementary Figure 3 — Kaplan–Meier curve for long-term all-cause mortality in all patients presenting with AMI. [file Image_3.TIFF]
