# Supplementary material for: Prognostic Outcomes in Acute Myocardial Infarction Patients Without Standard Modifiable Risk Factors: A Multiethnic Study of 8,680 Asian Patients
Source: Front Cardiovasc Med. 2022 Mar 29;9:869168. doi: 10.3389/fcvm.2022.869168 (PMC9001931; doi:10.3389/fcvm.2022.869168)
Supplement: Supplementary Table 1 — Outcomes of SMuRF-less compared to SMuRF patients, in patients without previous heart failure or stroke. [file Table_1.docx]

**Supplementary Table 1.** Outcomes of SMuRF-less compared to SMuRF patients, in patients without previous heart failure or stroke.

|  | **Overall (N=8057)** | **SMuRF (N=7329)** | **SMuRF-less (N=728)** | **P-Value** |
| --- | --- | --- | --- | --- |
| All-cause mortality | 531 (6.6) | 448 (6.1) | 83 (11.4) | <0.001 |
| Cardiac related mortality | 476 (5.9) | 399 (5.4) | 77 (10.6) | <0.001 |
| Cardiogenic shock | 618 (7.7) | 524 (7.1) | 94 (12.9) | <0.001 |
| Stroke | 135 (1.7) | 119 (1.6) | 16 (2.2) | 0.206 |
| Heart failure | 853 (10.6) | 790 (10.8) | 63 (8.7) | 0.079 |
| 30-day readmission | 1051 (13.0) | 958 (13.1) | 93 (12.8) | 0.725 |

* Outcomes are presented as n (%).

SMuRF, standard modifiable risk factors
