# Supplementary material for: Prognostic Outcomes in Acute Myocardial Infarction Patients Without Standard Modifiable Risk Factors: A Multiethnic Study of 8,680 Asian Patients
Source: Front Cardiovasc Med. 2022 Mar 29;9:869168. doi: 10.3389/fcvm.2022.869168 (PMC9001931; doi:10.3389/fcvm.2022.869168)
Supplement: Supplementary Table 2 — Outcomes of SMuRF-less compared to SMuRF patients, in patients without previous heart failure, stroke, or chronic kidney disease. [file Table_2.docx]

**Supplementary Table 2.** Outcomes of SMuRF-less compared to SMuRF patients, in patients without previous heart failure, stroke or chronic kidney disease.

|  | **Overall (N=7404)** | **SMuRF (N=6700)** | **SMuRF-less (N=704)** | **P-Value** |
| --- | --- | --- | --- | --- |
| All-cause mortality | 436 (5.9) | 359 (5.4) | 77 (10.9) | <0.001 |
| Cardiac related mortality | 390 (5.3) | 317 (4.7) | 73 (10.4) | <0.001 |
| Cardiogenic shock | 539 (7.3) | 447 (6.7) | 92 (13.1) | <0.001 |
| Stroke | 123 (1.7) | 108 (1.6) | 15 (2.2) | 0.253 |
| Heart failure | 666 (9.0) | 606 (9.0) | 60 (8.5) | 0.668 |
| 30-day readmission | 946 (12.8) | 856 (12.8) | 90 (12.8) | 0.938 |

* Outcomes are presented as n (%).

SMuRF, standard modifiable risk factors
