# Supplementary material for: Prognostic Outcomes in Acute Myocardial Infarction Patients Without Standard Modifiable Risk Factors: A Multiethnic Study of 8,680 Asian Patients
Source: Front Cardiovasc Med. 2022 Mar 29;9:869168. doi: 10.3389/fcvm.2022.869168 (PMC9001931; doi:10.3389/fcvm.2022.869168)
Supplement: Supplementary Table 3 — Multivariate analysis of the 30-day cardiovascular mortality between SMuRF and SMuRF-less. [file Table_3.docx]

**Supplementary Table 3. Multivariate analysis of 30 day Cardiovascular Mortality between SMuRF and SMuRF-less.**

|  | **HR** | **95% CI** | **P-value** |
| --- | --- | --- | --- |
| SMuRF-less | 1.480 | 1.090-1.860 | 0.048 |
| Age | 1.069 | 1.057-1.081 | <0.001 |
| Male | 0.906 | 0.662-1.240 | 0.536 |
| Ethnicity |  |  |  |
| *Malay* | 1.401 | 1.004 -1.956 | 0.048 |
| *Indian* | 1.500 | 1.044 -2.154 | 0.028 |
| *Caucasian* | 0.617 | 0.101-3.758 | 0.600 |
| *Chinese* | Reference |  |  |
| NSTEMI | 0.356 | 0.252-0.505 | <0.001 |
| Chronic kidney disease | 2.246 | 1.584-3.185 | <0.001 |
| Cardiac arrest | 15.492 | 11.452-20.957 | <0.001 |
| Left main/LAD disease | 1.404 | 1.079-1.827 | 0.012 |

Legend: HR – hazard ratio, CI – confidence interval, ACS – acute coronary syndrome, NSTEMI – non-ST elevation myocardial infarction, LAD – left anterior descending,
