# Supplementary material for: Prognostic Outcomes in Acute Myocardial Infarction Patients Without Standard Modifiable Risk Factors: A Multiethnic Study of 8,680 Asian Patients
Source: Front Cardiovasc Med. 2022 Mar 29;9:869168. doi: 10.3389/fcvm.2022.869168 (PMC9001931; doi:10.3389/fcvm.2022.869168)
Supplement: Supplementary Table 4 — In-hospital cardiovascular mortality assessed in the Fine-Gray model to account for competing risk. [file Table_4.docx]

**Supplementary Table 4. In-hospital cardiovascular mortality assessed in the Fine-Gray model to account for competing risk.**

|  | **SHR** | **95% CI** | **P-value** |
| --- | --- | --- | --- |
| SMuRF-less | 1.548 | 1.074-2.231 | 0.019 |
| Age | 1.064 | 1.052-1.076 | <0.001 |
| Gender | 1.043 | 0.778-1.399 | 0.778 |
| Ethnicity |  |  |  |
| *Malay* | 1.343 | 0.981-1.839 | 0.066 |
| *Indian* | 1.333 | 0.938-1.895 | 0.109 |
| *Caucasian* | 0.525 | 0.082-3.341 | 0.495 |
| *Chinese* | Reference |  |  |
| NSTEMI | 0.402 | 0.295-0.550 | <0.001 |
| Chronic kidney disease | 2.577 | 1.866-3.560 | <0.001 |
| Cardiac arrest | 14.534 | 10.799-19.562 | <0.001 |
| Left main/LAD disease | 1.266 | 0.988-1.624 | 0.063 |

Legend: SHR – sub-distribution hazard ratio, CI – confidence interval, ACS – acute coronary syndrome, NSTEMI – non-ST elevation myocardial infarction, LAD – left anterior descending,
